# Intravesicular Solute Delivery and Surface Area Regulation in Giant Unilamellar Vesicles Driven by Cycles of Osmotic Stresses

**DOI:** 10.1021/jacs.3c11679

**Published:** 2024-01-24

**Authors:** Pallavi D. Sambre, James C. S. Ho, Atul N. Parikh

**Affiliations:** † Department of Materials Science and Engineering, University of California, Davis, One Shields Avenue, Davis, California 95616, United States; ‡ Singapore Centre for Environmental Life Sciences Engineering, Nanyang Technological University, 59 Nanyang Drive, 636921 Singapore; § Institute for Digital Molecular Analytics and Science, Nanyang Technological University, 60 Nanyang Drive, 637551Singapore; ∥ Department of Biomedical Engineering, University of California, Davis, One Shields Avenue, Davis, California 95616, United States

## Abstract

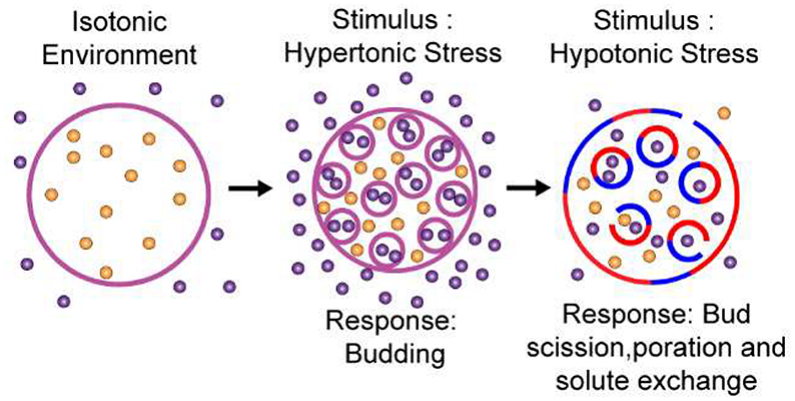

Phospholipid bilayers are dynamic cellular components that undergo constant changes in their topology, facilitating a broad diversity of physiological functions including endo- and exocytosis, cell division, and intracellular trafficking. These shape transformations consume energy, supplied invariably by the activity of proteins. Here, we show that cycles of oppositely directed osmotic stresses—unassisted by any protein activity—can induce well-defined remodeling of giant unilamellar vesicles, minimally recapitulating the phenomenologies of surface area homeostasis and macropinocytosis. We find that a stress cycle consisting of deflationary hypertonic stress followed by an inflationary hypotonic one prompts an elaborate sequence of membrane shape changes ultimately transporting molecular cargo from the outside into the intravesicular milieu. The initial osmotic deflation produces microscopic spherical invaginations. During the subsequent inflation, the first subpopulation contributes area to the swelling membrane, thereby providing a means for surface area regulation and tensional homeostasis. The second subpopulation vesiculates into the lumens of the mother vesicles, producing pinocytic vesicles. Remarkably, the gradients of solute concentrations between the GUV and the daughter pinocytic vesicles create cascades of water current, inducing pulsatory transient poration that enable solute exchange between the buds and the GUV interior. This results in an efficient water-flux-mediated delivery of molecular cargo across the membrane boundary. Our findings suggest a primitive physical mechanism for communication and transport across protocellular compartments driven only by osmotic stresses. They also suggest plausible physical routes for intravesicular, and possibly intracellular, delivery of ions, solutes, and molecular cargo stimulated simply by cycles of osmotic currents of water.

## Introduction

An astounding diversity of cellular activity involves folding, fusion, and fission of cellular membranes.^1,2^ Endocytosis—a mechanistically diverse and tightly regulated process by which macromolecules, nutrients, and pathogens from extracellular space enter the cell—proceeds through invagination and scission of the plasma membrane producing membrane vesicles (60–120 nm) inside the cytoplasm.^3^ Microautophagy—one of two pathways by which cytoplasmic components destined for degradation are directly taken up by the lysosome—invaginates the membrane of the lytic organelle into a bud, which is ultimately cleaved to produce macroautophagic bodies into the lysosomal lumen.^4^ The biogenesis of multivesicular endosomes (MVEs)—the transport intermediate between early and late endosomes, which sequester and deliver internalized receptors, nutrients, and ligands to late endosomes for digestion—similarly involves the inward budding of the cargo-rich segments of the endosomal membranes to accumulate 60–80 nm intraluminal vesicles (ILVs).^5−7^ Other topologically equivalent processes include the budding of enveloped viruses (50–200 nm) out of the cytosol, either from the cell surface or from endosomes,^8^ and secretion of exosomes (30–100 nm), resulting from the direct fusion of MVEs with the plasma membrane releasing the ILVs to the extracellular environment as extracellular vesicles.^9^ These phenomena, although regularly observed at nanoscales, are not pinned to it, readily extending to larger microscopic scales in processes like macropinocytosis (0.5–10 μm)^10^ and phagocytosis (1–3 μm)^11^—endocytic mechanisms by which cells ingest larger particles and take big gulps of fluids, respectively, that rely on the folding and the fission of the plasma membrane but at these larger, microscopic length scales.^12^

In all of these cases and several others, cellular membranes are dynamically remodeled. They are sculpted to produce and sever buds—both the protruding and the invaginating kinds—through a two-step sequential remodeling, one involving a morphological transition and the other a topological one.^13−15^ The initial morphological transition involves a curvature-driven bending of the flat membrane^2^ and constriction, which matures the invagination into an essentially spherical bud attached to the mother membrane by a thin neck. The subsequent topological transition proceeds via scission, breaking the material contiguity of the mother membrane and pinching off (or popping off) a complete vesicle.

These energy-intensive mechanical processes are seldom spontaneous. A crude estimate for the free-energy cost of bending an essentially flat membrane into a spherical bud can be obtained from the Helfrich model for local bending energy per unit area:^16^, where κ represents the bending modulus, *R*_1_ and *R*_2_ are the local radii of curvatures, and *R*_0_ represents the spontaneous curvature, which is a measure of asymmetry, either in composition or area or the environments, between the two leaflets.^17,18^

Thus, for a symmetric lipid bilayer (*R*_0_ = 0), the cost of creating a spherical vesicle (*R*_1_ = *R*_2_ = *R*) is simply . For a typical phospholipid bilayer (κ = 10–25 κ_B_*T*), this then suggests that the formation of a single spherical vesicle incurs a significant, size-independent free energy cost of 250–650 κ_B_*T* (where κ_B_*T* ≈ 0.6 kcal mol^–1^ represents the thermal energy)^19^—far exceeding typical binding energies of single proteins binding to lipid membranes.^20^ Thus, the large-scale membrane remodeling, which underscores the diverse variety of cellular activity above, inevitably requires cooperative interactions driven by the tightly regulated and synergistic activities of proteins,^21^ often augmented by additional membrane- or actin-mediated collective processes.^20^ For example, microautophagy, secretion of exosomes, and budding of viruses are fueled by the catalytic activities of ESCRT (endosomal sorting complex required for transport) family of proteins,^22−24^ which consume ATP.^25^ Endocytosis, in this vein, employs a variety of well-differentiated proteins, dependent on the type of cargo. The uptake of large particles (or cells >1 μm)—phagocytosis and macropinocytosis—draws the energy needed for membrane remodeling from the cooperative activities of cell surface receptors, membrane lipids, and actin cytoskeleton,^26,27^ whereas pinocytosis is driven by the actions of coat proteins^28^ such as clathrin^29^ or caveolin.^30^ In most endocytic routes, the membrane constriction and scission are performed by dynamin and dynamin-related proteins—a class of large (100 kDa) GTPases.^31,32^ These mechanochemical enzymes assemble as helical rings around the neck of the bud, constricting the neck and pinching off or popping off the bud from the mother membrane.^33,34^ Additionally, there are many cases of clathrin- and dynamin-independent routes,^2,35^ which engage the lipid machinery exploiting membrane elasticity (i.e., spontaneous curvatures) and their capacity for phase separation to drive budding and fission.^2,36,37^

Can this extraordinary mechanochemical feat—directed invagination, cargo selection, and compartmentalized uptake—be recapitulated in minimal synthetic cell-like compartments without the aid of the sophisticated protein machinery? Such an ability, we envisage, can endow synthetic cells with an ability to exchange information (and cargo) with their local environment, thereby providing a generic means for endocytosis. It may also yield critical information regarding how primitive cells might have met this essential requirement without the availability of complex protein machineries. In the work described here, we report that mere exposure to cycles of osmotic currents of water is sufficient to deform, divide, and fuse vesicular membranes in manners that allow for both the intravesicular exchange of solutes and the mimicking of the essential phenomenology of the surface area regulation.

## Results and Discussion

We begin by preparing giant unilamellar vesicles (GUVs, 10–50 μm in diameter), both single- and multicomponent, in sucrose solution (*C*_int_ = 100 mM) using the standard electroformation technique^38^ (see the Methods section). For single-component GUVs, we use a fluid-phase phospholipid, namely, 1-palmitoyl-2-oleoyl-*sn*-1-glycero-3-phosphocholine (POPC). Our multicomponent GUVs are composed of an equimolar mixture of POPC, cholesterol (Ch), and sphingomyelin (SM). Depending on the temperature, membrane mechanical tension, and solution conditions, bilayers of this lipid composition form a single uniform phase or undergo microscopic phase separation, with the latter characterized by two coexisting liquid phases: a dense phase enriched in SM and Ch designated as the *L*_*o*_ (liquid-ordered) phase and a second, less dense *L*_*d*_ (liquid-disordered) phase consisting primarily of POPC.^39^ To enable visualization of the membrane by fluorescence microscopy, the GUVs are doped with a small concentration of a probe lipid, namely 1,2-dioleoyl-*sn*-glycero-3-phosphoethanolamine-*N*-(lissamine rhodamine B sulfonyl) (Rho-DOPE, 1.0 mol %). For GUVs consisting of phase-separating lipid mixtures, we also doped the membrane with a second fluorescent probe, 1,2-dioleoyl-*sn*-glycero-3-phosphoethanolamine-*N*-(7-nitro-2-1,3-benzoxadiazol-4-yl) (ammonium salt) (NBD-PE, 3 mol %), which exhibits a greater preference for partitioning into the *L*_*o*_ phase.^40^ The sucrose-laden GUVs so prepared are then transferred to an osmotically balanced bath containing an isomolar (100 mM**)** concentration of less-dense glucose. The resulting density contrast gravitationally settles the GUVs onto the underlying glass.

### Exposure to a Hyperosmotic Environment Induces Characteristic Spherical Invagination in GUVs

Elevating the glucose concentration in the extravesicular bath to higher osmolarity (*C*_ext_ = 140 mM) subjects the GUVs to hypertonic stress: Δπ = *RT*Δ*c* ≈ 0.1 MPa (where *R* is the gas constant, 0.082 L atm K^–1^ mol^–1^; *T* is the temperature, K; and Δ*c* is the concentration gradient, 40 mM). As a result, water flows out of the vesicle reducing the osmotic pressure differential with a fast permeation time,^41^. Consequently, the vesicular volume (*V*) decreases abruptly in the fixed membrane area. Quantified in terms of reduced volume, , where *R*_0_ represents the initial radius of the undeformed GUV; this scenario (*v* < 1) then sets the stage for the membrane to deform. These excess area-induced shape deformations for large reduced volumes (*v* > 0.5) are known to produce a wide variety of theoretically predicted and experimentally validated axisymmetric shapes—including oblate, prolate, starfish, dumbbells, and pearls—at the new equilibria, each minimizing the membrane bending energy for the corresponding reduced volume (*v*).^42,43^

**Figure 1:**
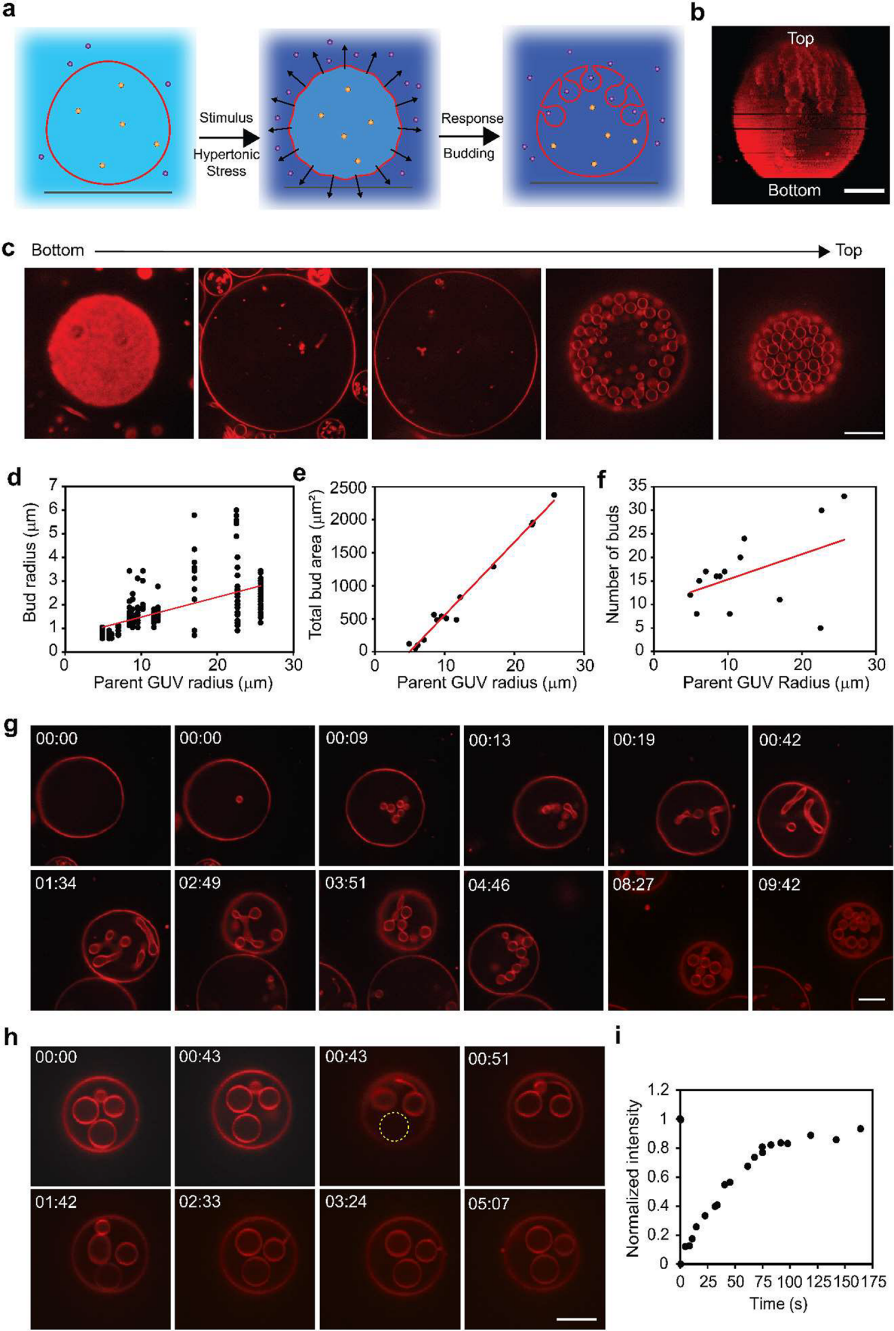
Hyperosmotic environment induces spherical invagination in giant unilamellar vesicles (GUVs). (a) Schematic representation of the invagination process stimulated by hypertonic stress (0.1 MPa). Arrows indicate the directions of water flux. Purple circles represent glucose, and orange represent sucrose. (b) A 3D stack and (c) 2D images from a time-lapse movie (Video S1) of confocal fluorescence microscopy images of GUVs. (d–f) Plots of (d) bud radius (*n*_bud_ = 232, where *n*_bud_ is the number of buds). (e) Total bud area (*n*_bud_ = 232). (f) Number of buds as a function of parent GUV radius (*n*_GUV_ = 14 GUVs, where *n*_GUV_ is the number of GUVs). Individual data points are plotted. (g) Selected frames from a time-lapse movie (Video S2) of GUVs subjected to hypertonic stress. (h, i) Fluorescence recovery after photobleaching (FRAP) measurement of a bud. (h) Selected frames from the time-lapse recording of the FRAP measurement (Video S2). (i) Representative fluorescence intensity profile of the photobleached bud (yellow dashed circle) (*n* = 19). All GUVs consist of 99 mol % POPC and 1 mol % Rho-DOPE, encapsulating sucrose (*C*_int_ = 100 mM) and are subjected to a hypertonic stress (0.1 MPa) (*C*_ext_ = 140 mM glucose). Scale bars: 10 μm.

In a stark departure from these predictions, the osmotically deflated GUVs in our experiments feature a well-defined and reproducible deformation in the steady state. For a range of reduced volumes, *v*∼ 0.5–0.9 (Δ*c* = 10, 40, and 100 mM), the height-resolved, confocal fluorescence microscopy slices reveal a highly polarized shape characterized by a flattened basal interface at the substrate surface and a strongly invaginated distal hemisphere consisting of a dense constellation of spherical invaginations or buds (Figure 1a–c and Video S1). The invaginating buds are essentially spherical; taut, exhibiting little or no thermal undulation of their membranes; and nearly monodisperse, measuring an average radius of 2.0 (±1.3 S.D.) μm for single-component POPC vesicles (Figure 1d) and 2.0 (±0.7 S.D.) μm for ternary composition vesicles (Figure S1). A representative GUV, on average, displays 17 (±8, S.D.) spherical invaginations for POPC GUVs (Figure 1f) and 18 (±9, S.D.) spherical invaginations for multicomponent GUVs (Figure S1) in the distal hemisphere.

Time-resolved measurements using spinning disc confocal fluorescence microscopy shed additional light on the pathways by which the membrane deforms. After hypertonic stress (0.1 MPa) is experienced, the POPC GUVs deflate instantaneously. The initially taut membrane boundary is quickly replaced by a flaccid, vigorously fluctuating one (Figure 1g and Video S2). This appearance of thermally excited undulation fluctuations is consistent with the reduced membrane tension, a consequence of the excess membrane area generated by the reduction in the vesicular volume. The membrane undulations iron out within the next several tens of seconds, accumulating excess surface area and folding them into several randomly dispersed spherical projections directed toward the interior of the GUVs. These measurements reveal that the buds do not preferentially appear in the distal hemisphere but rather appear randomly across the membrane surface.

With time, some (not all) of these spherical invaginations fuse with one another, create branched multibud networks, and occasionally widen, creating a diversity of invaginations consisting of isolated spherical buds in coexistence with extended multispherical morphologies that resemble pearled-tube morphologies. The multispherical invaginations have average diameters in the microscopic range (2.3 ± 0.6 μm, S.D., for 12 randomly selected invaginations) and often percolate inside the vesicular lumen approaching the diameter of the mother GUV. Curiously, they do not coarsen into uniform cylinders but rather stabilize as extended networks of bead-on-string-like structures. Over time, these multibud extensions, together with unfused single buds, preferentially migrate to the distal hemisphere of the mother GUV decorating the upper hemispheres of GUVs (Figure 1b,c,g and Video S2). Thus, the dense constellation of invaginated buds we observe consists of both single isolated buds and clusters of buds fused to one another in networks of multispherical buds^44,45^

The qualitative shape changes we observe and their corresponding trajectories are fully reproducible for a variety of membrane-forming amphiphiles and the mixtures we assessed (Table S1). The GUVs composed of (1) single component POPC (*n* = 31, where *n* refers to the number of experiments); (2) ternary lipid mixtures containing equimolar concentrations of cholesterol, POPC, and sphingomyelin (*n* = 25) (Video S3 and Figure S2); and (3) three-component, hybrid polymer–lipid GUVs composed of mixtures of polybutadiene (PBD)–poly(ethylene oxide) (PEO) amphiphilic block copolymer (PBD_22_-*b-* PEO_14_), cholesterol, and sphingomyelin (*n* = 3) (Video S4 and Figure S3) all exhibit comparable budding behaviors. These shape changes are fully reproducible for a variety of osmotic differentials (Δ*c* = 10, 40, and 100 mM) (Figure S4). Interestingly, the average sizes of the daughter vesicles (∼2 μm) appear to be largely constant, independent of the membrane composition or the magnitudes of the concentration gradients.

There are many salient features of this emergent membrane morphology that are particularly noteworthy. These are discussed in turn as follows.

*First*, the GUVs at the substrate surface flatten to assume the global shape of truncated spheroidal caps. In the limit of weak and nonspecific adhesion, such as in the present case, the settled GUVs spread at the substrate surface suppressing free membrane undulations (Video S5 and Figure S5) without introducing significant membrane tension. Here, the loss of entropic surface fluctuations is compensated by the gain of adhesion energy,^46−48^ and the GUV remains stably bound to the substrate surface—albeit weakly, nonspecifically, and in a laterally mobile manner, consistent with previous reports.^48,49^

*Second*, the appearance of spherical and multispherical bud morphologies upon osmotic deflation is a qualitative departure from typical stationary shapes derived through osmotic deflation of spherical GUVs.^17^ Morphologically comparable membrane vesiculation upon osmotic contraction has been previously reported, albeit under many different experimental conditions and specialized constraints.^50−53^ These studies identify several different mechanisms leading to membrane invaginations. These include (1) buckling or wrinkling instability of the membrane induced by the osmotic stress;^53^ (1) increased persistence lengths ξ_p_ of membranes, such as those containing cholesterol,^52^ which modulates the bending penalty to budding in a length scale dependent manner; (3) the interplay of substrate adhesion energy and membrane fluctuations, which spatially localizes surplus membranes as folds in the vicinity of the substrate surface;^50^ and (4) changes in electrostatically mediated surface adhesion, such as via dynamic exchange of cations (Ca^2+^).^51^

The most general unifying framework for understanding osmotically induced membrane invaginations invokes reduced volume, such as occurs during osmotic deflation, and spontaneous curvature, which arises because of imposed solution asymmetry surrounding the membrane.^44,54^ The osmotic deflation implies that the GUVs lose intravesicular volume in the fixed membrane area. As a consequence, the newly created excess membrane area undergoes reorganization to minimize the free energy.^17^ Geometrically, the inward budding (as opposed to the outward budding) is much more efficient at compensating for the volume loss while folding the excess membrane.^52^ Second, the sucrose-encapsulating GUVs coming in contact with the external bath containing glucose now experience trans-bilayer asymmetry and thus acquire a preferred nonzero spontaneous curvature.^17,55^

The formation of spherical invaginations is consistent with the appearance of a spontaneous curvature. For an average spherical bud, ∼2 μm in diameter, the spontaneous curvature generated is 0.5 μm^–1^. This value must exceed the threshold predicted from curvature elasticity theories,^56,57^ which depend on the GUV volume and the membrane area. Moreover, because the buds produced invariably extend toward the interior lumen of the mother GUVs, the generated spontaneous curvature, presumably because of the asymmetry induced by the differences in the dissolved solutes, must also be negative. A recent study, however, suggests that the lipid bilayers experiencing glucose–sucrose asymmetry tend to bend in the direction of the glucose-laden solution,^54^ which in our case would produce outward budding. In our case, it is clear that both the volume reduction and spontaneous curvature play a role. Additional experiments would be needed to fully validate these notions.

*Third*, the invaginated buds are connected to the mother membrane. Photobleaching fluorescent membrane probes (Rho-DOPE) in single buds (Figure 1h) and monitoring their recovery reveal that the lipids in the bud are in fluid contiguity with those in the mother membrane (Figure 1i and Video S6). This observation remains consistent for ternary vesicle compositions (Video S7 and Figure S6).

**Figure 2:**
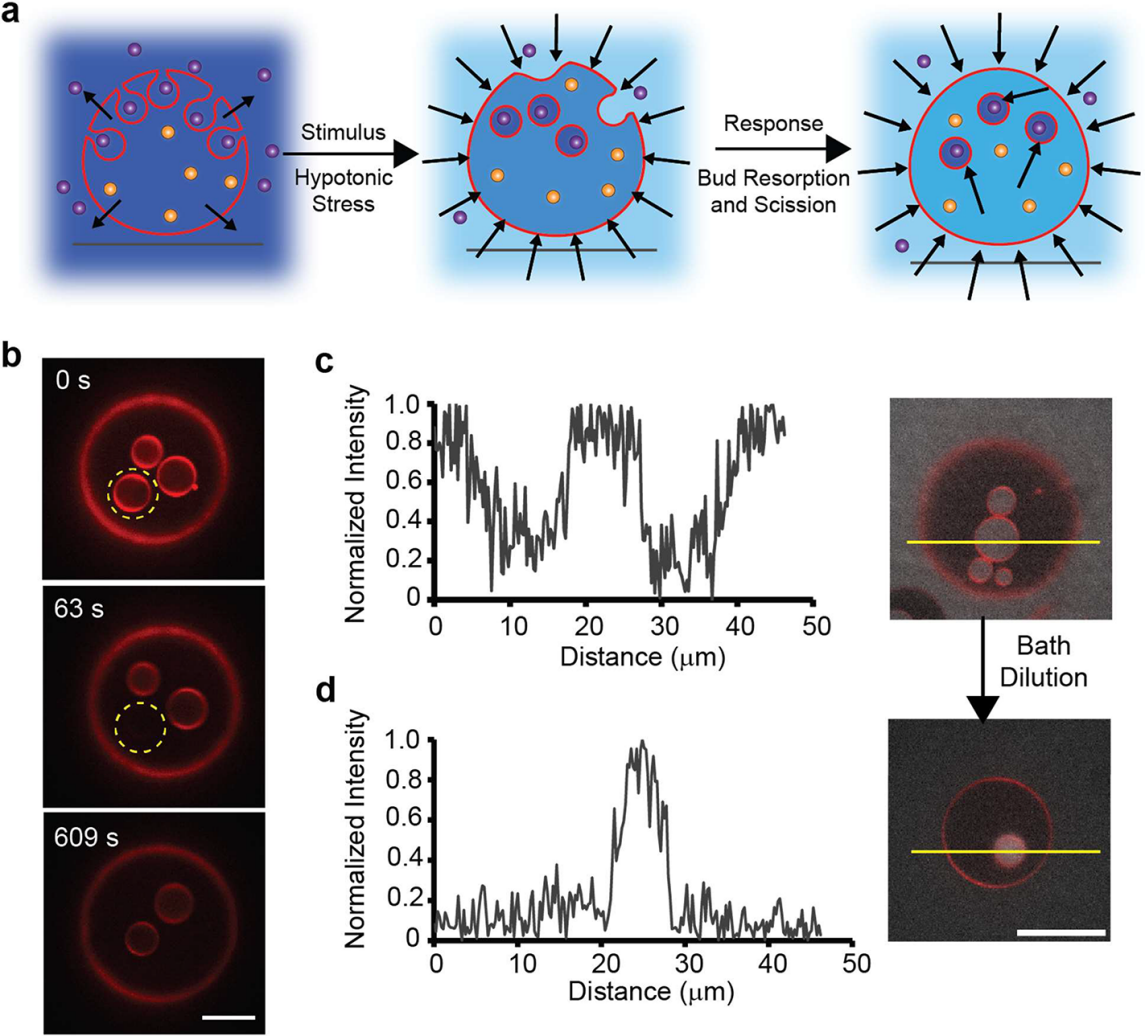
Osmotic cycling of single-component vesicles. (a) Schematic representation of the morphological changes of a vesicle subjected to a sequential two-step osmotic cycling process. Arrows indicate the water flux direction. Purple circles represent glucose, and orange circles represent sucrose. (b) Selected frames from a time-lapse recording of fluorescence recovery after photobleaching (FRAP) measurement (Video S8) (*n* = 9). Scale bar: 10 μm. (c, d) Vesicles subjected to the two-step osmotic cycle using fluorescently doped glucose solution (0.1 mM NBD-glucose) in the exterior bath. Plots of intensity profiles (left panel) of the yellow lines (right panel) overlaid on representative fluorescence micrographs from the (c) Step 1 and (d) Step 2 osmotic cycling process (*n* = 3). All GUVs consist of 99 mol % POPC and 1 mol % Rho-DOPE, encapsulating sucrose (*C*_int_ = 100 mM) and are subjected to a hypertonic stress (0.1 MPa) (*C*_ext_ = 140 mM glucose). Scale bar: 20 μm.

*Fourth*, the invaginated volume does not compromise the vesicular compartmentalization of the aqueous phase: the outside and the inside do not mix. Simply adding trace concentrations of fluorescently labeled, water-soluble markers (e.g., NBD-labeled glucose, 0.1 mM) to the extravesicular surroundings reveals that the volumes enclosed by the invagination are contiguous with the external bath and remain fully unmixed with the intravesicular milieu. This then establishes that the topological contiguity and compartmental integrity of the mother vesicle are not compromised during these osmotically induced deformations (Figure 2c).

*Fifth*, it is notable that the buds accumulate at the distal hemisphere, away from the bounding surface, similar to what has previously been observed.^53^ In view of the uncompromised compartmentalization, this is not surprising: The buds generated by the GUV deformation engulf the glucose-laden (0.99 g/cm^3^, 140 mM aqueous solution) external solution. As a result, they are lighter dents or inverted cups interfacing with the denser sucrose environment (1.03 g/cm^3^, 140 mM aqueous solution) of the GUV interiors. This density contrast, we speculate, is sufficiently large that it can push the less dense glucose encapsulating buds to rise and cluster toward the distal hemisphere. This, together with the flattened proximal surface, creates a strongly polarized vesicular morphology (Figure 1b,c).

*Sixth*, the formation of the daughter buds pulls lipids from the surface of the mother membrane. Because the membrane is essentially incompressible, this then requires that the size of the mother membrane decrease with the appearance of invaginated buds. This requirement for the conservation of the membrane area yields = 4*πR*^2^ + *n*4*πr*^2^, where *R*_0_ is the initial radius of the mother GUV; *R*, the radius of the final GUV; *r*, the radius of the spherical bud; and *n*, the number of daughter buds. This simple geometric balance for a typical POPC GUV (*R*_0_ ∼ 20 μm), which has shrunk to *R* ∼17 μm, then predicts ∼27 (*r* ∼ 2 μm) buds, comparable to our experimental observations (Figure 1e–g).

### Membrane Area Homeostasis Facilitated by a Subpopulation of Invaginations

In the cellular context, membrane invaginations are common features characterizing the topography of the plasma membrane of many cells.^58−60^ A particularly interesting example is the class of membrane invaginations called caveolae: 60–80 nm bulb-shaped surface invaginations or pits that contain oligomeric caveolin and are enriched in cholesterol and sphingolipids. Recent theoretical and experimental studies suggest that these invaginations play important roles as sensors and regulators of membrane tension:^61,62^ In response to acute mechanical stresses, such as those produced by osmotic swelling or uniaxial stretches, the invaginations flatten and disassemble. They thus act as mechanosensitive membrane reservoirs that passively buffer against changes in membrane tension^63,64^ by acting as readily available sources of the extra membrane surface. They thus provide a more efficient route for surface area regulation (SAR), wherein excess “endomembranes” buffer against tensional deviations by adding or depleting membrane surface area—an otherwise slower process.^64^ But the evidence is also mounting in support of caveolae fission under tension. Here, caveolae do not resorb into the plasma membrane but rather bud off and endocytose to produce caveosomes, which subsequently transform into early endosomes inside the cytoplasm.^65^ The morphological similarities between the vesicular invaginations we observe and cell surface caveolae suggest several interesting possibilities: Do osmotically generated, spherical invaginations, such as we observe here, participate in surface area regulation? Do they reincorporate within the mother membrane? Do they detach, becoming endocytosed within the vesicular lumen? Do these membrane pits thus transport extravesicular content to the inside? Once inside, do they remain intact?

To address these questions, we subject GUVs to a two-step osmotic cycle: Step 1 subjects the GUVs encapsulating sucrose (*C*_int_ = 100 mM) to hypertonic stress (0.1 MPa) (*C*_ext_ = 140 mM) as above. Step 2 reverses the direction of the osmotic gradient by subjecting the products of Step 1 to a hypotonic bath (*C*_ext,2_ = 60 mM) (Step 2). Note that the osmotic equilibrium in Step 1 implies that the vesicular volume adjusts during Step 1 to drive the sucrose concentration inside to match the glucose concentration outside (*C*_ext_ = 140 mM). Thus, Step 2 subjects the invaginated GUVs to a concentration differential (Δ*c* = 60–140 = −80 mM) and a net hypotonic stress, Δπ = *RT*Δ*c* ≈ 0.2 MPa.

Imaging a representative GUV sample after Step 2 using fluorescence microscopy, two quantitative changes to the morphologies generated by Step 1 are evident: (1) reduction in the average number of buds and (2) increase in the size of the mother GUV. In a set of representative GUVs (*n*_GUV_ = 15), the bud numbers decreased from 17 ± 1 (after Step 1) to 6 ± 2 (after Step 2), and the average radius of the mother GUV increases by 10.6% (17.0 ± 1.0 μm) compared to the sizes obtained after Step 1 (15.4 ± 0.7 μm) (Figure S7a).

These two changes lend support to the notion that the buds act to buffer the mechanical tension and contribute to the membrane area homeostasis, as detailed below. The observed increase in the GUV sizes during Step 2 cannot be accounted for by considering membrane stretching alone. Previous studies have shown that membranes can stretch up to 2–6% in the area prior to lysis.^64,66^ Thus, the higher area increase we see suggests that the buds might serve as a source for extra membrane surface by resorbing into the mother membrane during inflation induced in Step 2. Moreover, the GUV sizes do not return to predeflation (prior to Step 1) values (17.0 ± 1.0 μm vs 17.7 ± 0.8 μm) (Figure S7a). This is not surprising because not all daughter buds are resorbed in our experiments; several buds remain within the lumen (see the Methods section). This is then consistent with a picture in which some of the buds flatten—buffering the hypotonic stress-induced swelling of the mother GUVs and contributing to GUV’s tensional homeostasis.^64^

### Endocytosis of a Subpopulation of Invaginated Buds

To further probe the fate of the buds that do not flatten, resorb, or contribute to osmotic relaxation, we repeat the two-step osmotic cycle using fluorescently doped glucose solution in the exterior bath, using a small concentration (0.1 mM) of NBD-glucose during Step 1. The GUVs as a result now contain NBD-glucose (green) along with the external solution entrapped within their newly formed buds. During Step 2, when the osmolarity of the external bath is lowered to (*C*_ext,2_ = 60 mM), the fluorescence intensity due to glucose decreases (∼80%) as expected. Strikingly, however, the volume of the fluid cupped by the invaginations reveals little or no change in the green fluorescence intensity (δ*I* = 0). This then suggests that the fluid from the exterior, which is engulfed by the bud, is physically isolated from the outside bath (Figures 2c,d and S8). There are two limiting scenarios that can help reconcile this seemingly counterintuitive observation: (1) the formation of a closed bud neck or (2) a topological transition cleaving the daughter bud from the mother GUV. In the closed-neck scenario,^17,67^ the neck restricts the passage of the fluorescent NBD-glucose from the altered outer solution inside the bud. It thus isolates the solution within the budded invaginations from the bulk exterior, thus preventing equilibration of the two. This scenario, however, suggests that the membrane remains contiguous. In the second scenario, the buds pinch off from the mother GUV through a topological transition, creating free-floating isolated vesicles within the mother GUVs, thus breaking the fluid contiguity with the external bath. A fluorescence recovery after the photobleaching (FRAP) measurement addresses this issue. Briefly, we photobleached a fluorescent probe lipid, Rho-DOPE, in the membrane of a bud within the lumen of the GUV after the osmotic cycle (Steps 1 and 2). Remarkably, we find little or no recovery of the bud membrane intensity (Figure 2b, Video S8, and Figure S9), confirming that the membrane of the invaginated bud is topologically isolated. In other words, the bud underwent complete fission, thereby producing a free-floating daughter vesicle within the lumens of the mother GUVs (Figure 2a).

These findings are remarkable for the fact that division of uniform, single-component vesicles, in general, is exceedingly difficult.^68−70^ Previous studies have shown that the free energy barrier, which arises from the local disruption of the bilayer motif, may be met by a combination of spontaneous curvature generation, which acts to lower the free-energy barrier and the presence of curvature-induced constriction forces^69,71^ acting on the neck connecting the bud to the mother. It is likely that during the second hypotonic stage of the cycle the rapid fluxes of water can accumulate such constriction forces at the neck. But this proposition remains unverified.

### Vesiculated Buds, Membrane Composition, and Cargo Transport

The osmotic cycling-induced budding and division observed above raises a curious question: How does the compositional diversity of the membrane influence the dynamics described above? In the cellular context, this simple question has important parallels. Cellular membranes are invariably compositionally heterogeneous. Moreover, many cellular substructures, specifically invaginations and pits such as caveolae and clathrin-coated pits, concentrate certain lipids and expel others.^58−60^ To investigate whether the osmotically triggered shape and topological transitions reported here dynamically activate membrane’s compositional degrees of freedom, we prepared GUVs using a ternary mixture containing an equimolar concentration of POPC, cholesterol, and sphingomyelin. This mixture is known to produce a uniform lipid bilayer phase in unstressed GUVs but transform into a phase-separated state when mechanically stretched.^72−74^ Subjecting these multicomponent GUVs to a hypertonic stress (0.1 MPa) (Δ*C* = 40 mM aqueous glucose solution) (Step 1), spherical invaginations accumulate near the distal boundary, fully consistent with the behaviors of single-component GUVs (see earlier section). Notably, the membrane surface remains uniform at the optical length scale, suggesting no large-scale lipid phase separation. We now subject the vesicles to a subsequent hypotonic challenge of Step 2 (Figure 2a). This exposes the invaginated GUVs, obtained at Step 1, to a hypotonic bath, Δ*C* = 80 mM, that introduces an inflating osmotic stress (0.2 MPa). Here too, some of the buds are reincorporated into the mother membrane, and others cleave away, producing free daughter vesicles within the GUVs (Figures S8 and S9).

**Figure 3:**
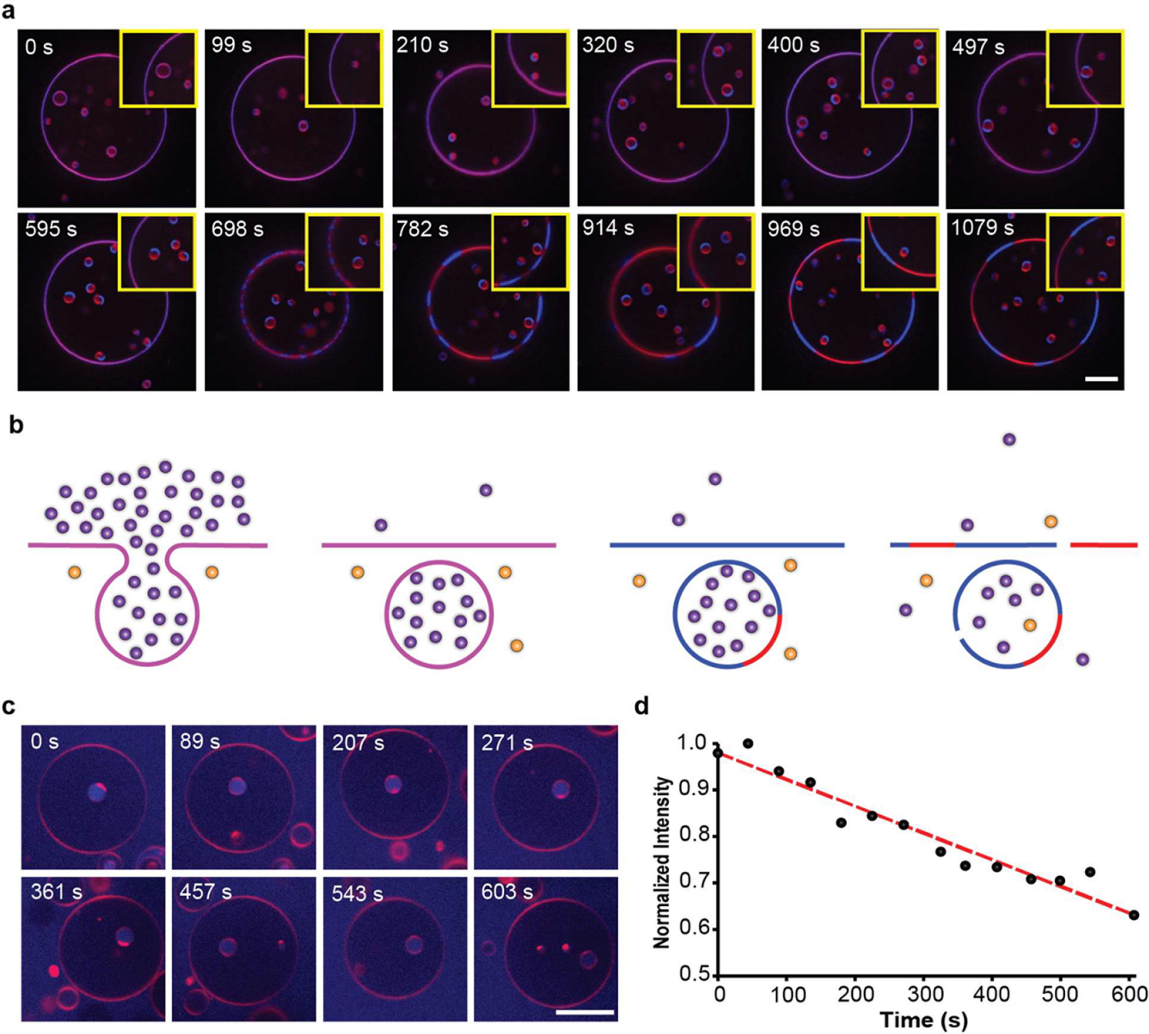
Osmotic cycling of ternary vesicles. (a) Selected frames from a time-lapse movie (Video S9) of ternary vesicles subjected to hypertonic stress. The GUV consists of 32 mol % POPC, 32 mol % Ch, 32 mol % SM, 1 mol % Rho-DOPE (red), and 3 mol % NBD-PE (blue) (*n* = 9). Scale bar: 10 μm. (b) Schematic representation of the invagination process stimulated a two-step osmotic cycling process for ternary GUVs. Purple circles represent glucose, and orange circles represent sucrose. (c) Selected frames from a time-lapse movie (intermittent snapshots to avoid extensive photobleaching artifacts)) of ternary vesicles subjected to the two-step osmotic cycling process (*n* = 11). The GUV consists of 33 mol % POPC, 33 mol % SM, 33 mol % Ch, and 1 mol % Rho-DOPE (red), subjected to the two-step osmotic cycle using fluorescently doped (0.1 mM NBD-glucose) glucose solution (blue) in the exterior bath. Scale bar: 20 μm. (d) Plot of mean fluorescence intensity of the bud interior (c) as a function of time.

Remarkably, we find that this osmotic cycling also induces gross, large-scale redistribution of membrane components for the ternary component vesicles (Video S9). Figure 3a documents a typical trajectory. At the onset of Step 2 (*t* = 0 s), we have a ternary component GUV, which is compositionally homogeneous at the optical length scale and polarized with a flattened interface at the substrate surface and densely decorated with spherical invaginations at the distal end. At this point, the membranes of both the mother GUV and the invaginated buds display uniform fluorescence intensities at the optical length scales. At roughly 210 s after the initiation of Step 2, this compositional uniformity is abruptly replaced by a pattern of fluorescence intensities for several of the invaginated buds. Marked by the demixing of fluorescent probe lipids (Rho-DOPE and NBD-PE)—which are known to preferentially partition with liquid disordered (*L*_*d*_) and liquid ordered phase (*L*_*o*_)^39^—this appearance of the Janus pattern reflects the lateral phase separation of the membranes of the daughter vesicles into two coexisting phases producing monodomain organization. Over the next several hundreds of seconds (∼360 s), this Janus patterning of daughter vesicles becomes a global feature, extending to all the buds within the mother GUV. Also note that during this entire period (210–595 s), the mother GUV membrane remains fluorescently uniform, suggesting little or no phase separation at the optical length scales. However, at about 600–700 s, interestingly, the membrane of the mother GUV also becomes optically phase separated. Unlike the bud, the membrane of the GUV phase separates into many discrete domains, which coalesce and fragment repeatedly over several hundreds of seconds, exhibiting extraordinary pulsatory dynamics.

This remarkable coupling of spatial organization of membrane molecules (and corresponding lateral phase separation) and the osmotic activity of water originates from a well-orchestrated synergy of several biophysical processes. After the Step 1 hypertonic challenge, the interior of the GUVs osmotically equilibrates to match the outside concentration (∼140 mM). The cleaved buds, which have become free-floating vesicles inside GUVs, also encapsulate the elevated concentration of extravesicular glucose (∼140 mM). The second osmotic relaxation triggered by the hypotonic challenge of Step 2 sets in complex fluxes of water across the two nested compartments: Water first enters the aqueous interior of the GUV diluting the lumen because of the applied osmotic pressure difference between the bath and the GUV interior (Δ*P*_osmotic_ = Δ*cRT*). This is opposed by the Laplace pressure . At equilibrium, the balance of two pressures implies that . The same process applies to the daughter vesicles. The influx of water in the mother GUV renders the daughter vesicles hypertonic. Thus, water rushes into the daughter vesicles (Figure 3a). As a result of these water fluxes, both mother GUV and daughter vesicle membranes swell, becoming mechanically tense. But because the daughter vesicles are smaller, they experience a smaller buildup of mechanical tension. Yet, we find that they phase separate readily prior to the mother membrane. This is likely because of compositional differences between the mother and the bud membranes, shifting the bud composition in the phase-separated region of the phase diagram.^39^ Such compositional differences between the bud and the mother are better exemplified in our three-component, hybrid polymer–lipid GUVs, where buds exclusively arise from the more bendable lipid phase (Video S4 and Figure S3).

The observations of osmotically induced swelling and lipid–lipid phase separation above are in excellent correspondence with the well-known swell-burst dynamics in giant vesicles subjected to hypotonic environment.^75,71^ Here, the osmotic influx of water stretches the membrane. The corresponding buildup of lateral membrane tension has been shown by us and others to promote isothermal phase transition from homogeneous state to the phase-separated state in multicomponent membranes,^74,76^ such as we observe here.

Furthermore, when vesicular membranes are stretched beyond a certain threshold tension,^77,78^ it is known that membrane poration and vesicle rupture becomes energetically favorable, lysing the vesicles.^79^ A previous theoretical model by Idiart and Levin^77,78^ predicts that concentration gradients of as little as 1 mM are sufficient to induce poration. Interestingly, the vesicle lysis occurs in a stepwise manner driven by a cascade of transient pores.^80−82^ During each membrane poration event, a small proportion of the intravesicular solute (and water) is released before the bilayer reseals, leaving the vesicle hyperosmotic with a lower osmotic differential. This then prompts subsequent events of water influx, vesicle swelling, and rupture until sufficient intravesicular solute has been lost, and the membrane is able to withstand the residual sublytic osmotic pressure without collapsing.^83^ These pulsatory response of hypertonic vesicles, characterized by pulsations in size, state of mixing, and membrane integrity, ultimately leads to stepwise osmotic equilibration.^76^

The considerations above lead naturally to a simple question: Do newly formed buds, which are hypertonic relative to the GUV (as well as the exterior), also undergo poration? If yes, might they provide a mechanism to transfer cargo from extravesicular space inside the GUVs (Figure 3b)?

To address these questions and better probe the movement of the aqueous solutions and solutes across the nested compartments, we doped the exterior glucose-laden aqueous solution with trace concentration of fluorescently labeled NBD-glucose (0.1 mM) during Step 1 of the two-step osmotic cycle. Consistent with the foregoing experiments, the invaginated spherical buds entrapping the fluorescently labeled exterior solution is immediately evident after Step 1 (Figure 3c). At this point, the interior of the pinched-off and vesiculated buds remains fully isolated from both the exterior aqueous phase and the interior of the mother GUV (Figures 3c and S8). Over time, however, the bud intensity displays a significant decay (∼40%) even after accounting for photofading due to repeated illumination (Figure 3d). Note that the observed gradual and continuous decline in the fluorophore concentration does not allow us to deduce whether the cargo release is effected by transiently opening and closing cascades of pores^80−82^ or a continuous open pore. But a transient pore-based release would be consistent with many previous observations of swell-burst cycles of hypertonic vesicles.^76^ In other words, the vesiculated buds successfully transport into the GUV interior the molecular cargo they trafficked from the vesicular exterior, in an extraordinary functional resemblance to the process of macropinocytosis. Note that the cargo delivery process does not require phase separation or multicomponent membranes. Single-component GUVs composed of POPC alone yield qualitatively comparable solute delivery (Figure S10).

## Conclusion

Taken together, the results reported herein can be reconciled in terms of a single unifying mechanistic picture. As prepared, GUVs filled with a sucrose solution are surrounded by an isotonic glucose solution at the same average concentration. Consequently, the GUV membrane is mechanically unstressed and compositionally mixed for ternary compositions, at least at the optical length scales. When subjected to a stress cycle consisting of deflationary hypertonic stress (Step 1) followed by inflationary hypotonic stress (Step 2), the GUVs bud, vesiculate, porate, and deliver content from the extravesicular environment into the intravesicular milieu. The exposure to hypertonic stress in Step 1 deflates the GUVs. The excess membrane area, so created, folds into spherical invaginations, which buoy to the apex producing a dense collection of spherical invaginations at the distal end of the surface settled GUVs. During Step 2, the influx of water remodels the invaginations. A subpopulation participates in contributing area to the swelling membrane, thereby providing a mechanism for surface area regulation, a component of membrane area homeostasis. The second subpopulation, by contrast, exploits the hydrodynamic fluxes to sever from the mother membrane, vesiculating into the lumens of the mother vesicles. Remarkably, the gradients of solute concentrations so created by the nested compartments (i.e., mother GUV and the daughter vesiculated buds) create cascades of water current, which in turn induces pulsatory transient poration enabling solute exchange between the buds and the GUV interior. The net result then is an efficient water flux mediated delivery of molecular cargo across the membrane compartment in a manner functionally comparable to that of the cellular pinocytic processes.

Our findings suggest a primitive mechanism for communication and transport across protocellular compartments and their aqueous baths of rapidly fluctuating solute concentrations driven only by osmotically propelled water activity. They also suggest physical routes for intravesicular and possibly intracellular transport of ions, solutes, and molecular cargo in synthetic cells stimulated simply by cycles of osmotic currents of water.

## Materials and Methods

### Materials

1-Palmitoyl-2-oleoylglycero-3-phosphocholine (POPC), egg sphingomyelin (SM), cholesterol (Ch), and 1,2-dipalmitoyl-*sn*-glycero-3-phosphoethanolamine-*N*-(lissamine rhodamine B sulfonyl)(ammonium salt) (Rho-DOPE) were purchased from Avanti Polar Lipids (Alabaster, AL). Poly(1,2-butadiene)-*b*-poly(ethylene oxide) (PBD_22_-*b-*PEO_14_) with the average molecular weights of 1200 and 600 g/mol for the PBD and PEO blocks, respectively, was purchased from Polymer Source Inc. (Dorval, Quebec, Canada). Sucrose, glucose, and chloroform were purchased from Sigma-Aldrich (St. Louis, MO). *N*-(7-Nitrobenz-2-oxa-1,3-diazol-4-yl)-1,2-dihexadecanoyl-*sn*-glycero-3-phosphoethanolamine, triethylammonium salt (NBD-PE) and 2-(*N*-(7-nitrobenz-2-oxa-1,3-diazol-4-yl)amino)-2-deoxyglucose (2-NBDG) were purchased from ThermoFisher Scientific (Eugene, OR). Sucrose, glucose, and 2-NBDG solutions were prepared in deionized water (DI) (Millipore, Sigma, St. Louis, MO) were used for experimentation.

### Preparation of Vesicles

The lipids and polymer were dissolved in chloroform and mixed to different molar ratios to prepare giant unilamellar vesicles (GUV) of different compositions: (1) 99 mol % POPC and 1 mol % Rho-DOPE; (2) 32 mol % POPC, 32 mol % Ch, 32 mol % SM, 1 mol % Rho-DOPE, and 3 mol % NBD-PE; (3) 33 mol % POPC, 33 mol % SM, 33 mol % Ch, and 1 mol % Rho-DOPE; and (4) 32 mol % PBD_22_-*b-* PEO_14_, 32 mol % POPC, 32 mol % Ch, 1 mol % Rho-DOPE, and 3 mol % NBD-PE. Appropriate quantities (∼15 μL) of the above listed lipid mixtures were deposited onto two clean ITO glass slides and desiccated for at least 3 h. An O-ring while entrapping solution (100 mM sucrose) that hydrated the lipids during GUV formation was sandwiched between the desiccated slides, sealed with vacuum grease. GUV formation was conducted by adapting to a previously established electroformation method.^84^ The sandwich was subjected to 1.5 h of continuous sine wave current (10 Hz, 4 V_pp_) and 1.5 h of continuous square wave current (2 Hz, 4 V_pp_) to generate a high yield of 5–50 μm sized GUVs with excellent reproducibility. Electroformation was conducted at 45 °C for ternary composition vesicles and 55 °C for vesicles containing polymer, in accordance with the gel–fluid transition temperatures of the lipid or lipid/polymer mixtures. The electroformation current cycles were altered to 3 h of sine wave and 2 h of square wave for polymer composition.

### Budding of GUVs

One microliter of the prepared GUVs containing 100 mM sucrose was subjected 100 μL of 100 mM glucose bath in a 96-well plate. Introducing 25 μL of 300 mM glucose solution to the system changed the bath concentration to 140 mM glucose. This also resulted in excessive inward multivesiculations in the GUVs. Alternatively, directly subjecting 1 μL of GUVs (containing 100 mM sucrose) to a 140 mM glucose bath (hypertonic quench) gave rise to comparable results.

### Osmotic Cycling of the Budded Vesicles

The budded vesicles were subjected to hypotonicity by introducing 158.3 μL of DI water into the bath, making the bath molarity ∼60 mM.

### Imaging and Analysis

Vesicles were monitored in real time using a fluorescence microscope equipped with a spinning disk confocal configuration using an Intelligent Imaging Innovations Marianas Digital Microscopy Workstation (3i; Denver, CO) fitted with a CSU-X1 spinning disk head (Yokogawa Musashino, Tokyo, Japan) and a QuantEM512SC electron-multiplying charge-coupled device (EMCCD) camera (Photometrics, Tuscan, AZ). Fluorescence micrographs were obtained using oil immersion objective (60×, NA = 1.40 Plan Apo VC); Carl Zeiss Oberkochen, Germany). Rho-DOPE (Ex/Em; 560 nm/583 nm) was exposed with a 50 mW 561 nm laser line, and NBD-PE (Ex/Em; 460 nm/535 nm) and 2-NBDG (Ex/Em; 465 nm/540 nm) were exposed with a 50 mW 488 nm laser line. Fluorescence recovery after photobleaching (FRAP) experiments were performed with a 50 mW 561 nm laser line. Invaginations/buds were viewed with a 60× objective and a QuantEM512SC EMCCD camera, giving a 512 × 512 pixel image. Rho-DOPE fluorescent probes were bleached in a circular region (perimeter of the bud) at 100% of maximal laser power for 10 ms. Recovery of fluorescence was recorded and measured from the subsequent 3000 frames. The images are subsequently analyzed using ImageJ (http://rsbweb.nih.gov/ij/), a public-domain software, and Slidebook digital microscopy imaging software (3i Denver, CO).

### Dynamics of Intraluminal Buds

The isolated buds within the lumen of the mother GUV produced after the osmotic cycling (Steps 1 and 2) are highly dynamic. Some fuse with one another, others migrate out of the giant vesicle presumably through transient pores formed in the mother GUVs, and yet others simply burst (Figure S7b–d). All of these activities reduce the total number of buds in the GUV over time, even those that do not contribute to the osmotic relaxation at Step 2.

## Data Availability

Data supporting the findings of this study are available within this article and its Supporting Information.

## Supporting Information Available

Summary of the frequencies of reported events for each of the various experimental conditions studied; Video S1–S9 legends; statistical analysis of spherical invagination in ternary component vesicles; selected frames from time-lapse movie of ternary GUVs subjected to hyperosmotic stress; POPC vesicles subjected to different osmolyte concentration differences; control POPC vesicles at isotonic condition; FRAP for budded ternary component vesicles; morphological analysis of budded vesicles undergoing osmotic cycling process; solute entrapment analysis (PDF)
Video S1 (AVI)
Video S2 (AVI)
Video S3 (AVI)
Video S4 (AVI)
Video S5 (AVI)
Video S6 (AVI)
Video S7 (AVI)
Video S8 (AVI)
Video S9 (AVI)

## Supplementary Material

ja3c11679_si_001.pdf

ja3c11679_si_002.avi

ja3c11679_si_003.avi

ja3c11679_si_004.avi

ja3c11679_si_005.avi

ja3c11679_si_006.avi

ja3c11679_si_007.avi

ja3c11679_si_008.avi

ja3c11679_si_009.avi

ja3c11679_si_010.avi
