# Supplementary material for: Intravesicular Solute Delivery and Surface Area Regulation in Giant Unilamellar Vesicles Driven by Cycles of Osmotic Stresses
Source: J Am Chem Soc. 2024 Jan 24;146(5):3250–61. doi: 10.1021/jacs.3c11679 (PMC10859933; doi:10.1021/jacs.3c11679)
Supplement: Supplementary file 1 — ja3c11679_si_001.pdf [file ja3c11679_si_001.pdf]

SUPPLEMENTARY INFORMATION

Intravesicular Solute Delivery and Surface Area Regulation in Giant Unilamellar Vesicles Driven by Cycles of Osmotic Stresses

Pallavi D. Sambre[1], James C.S. Ho[2], and Atul N. Parikh[1,2,3]

[1] Department of Materials Science and Engineering, University of California, One Shields Avenue, California, 95616, USA

[2]Singapore Centre for Environmental Life Sciences Engineering and Institute for Digital Molecular Analytics and Science, Nanyang Technological University, Nanyang Technological University, 60 Nanyang Drive, 637551, Singapore

[3]Department of Biomedical Engineering, University of California, Davis, One Shields Avenue, Davis, California, 95616, USA

Contents:

Table S1	S2
Video S1-S9 Legends	S3
Figure S1-S10	S4-S11

Table S1. A summary of the frequencies of reported events for each of the various experimental conditions studied (see text for details).

Experiment	Total experiments	Positive experiments (n)	Probability of the reported event based on the no. of experiments (%)
**Total Osmocycling experiments with Step 1 at Δc=40 mM**			
POPC (Step 1)	32	31	96.9
POPC (Step 2)	17	16	94.1
POPC:SM:Ch (Step 1)	26	25	96.2
POPC:SM:Ch (Step 2)	34	31	91.2
**1. Osmocycling with fluorescently doped glucose solution**			
POPC (Step 1)	4	3	75
POPC (Step 2)	3	3	100
POPC:SM:Ch (Step 1)	8	7	87.5
POPC:SM:Ch (Step 2)	8	8	100
**2. FRAP**			
POPC (Step 1)	19	19	100
POPC (Step 2)	9	9	100
POPC:SM:Ch (Step 1)	8	8	100
POPC:SM:Ch (Step 2)	3	3	100
**3. Phase separation on Osmocycling**			
POPC:SM:Ch (Step 2)	9	9	100
**4. Solute transfer on Osmocycling**			
POPC (Step 2)	5	4	80
POPC:SM:Ch (Step 2)	14	11	78.6
**Hyperosmotic stress at different Δc**			
POPC (Step 1), Δc=10 mM	8	6	75
POPC (Step 1), Δc=100 mM	3	3	100

Video S1. Hyperosmotic induced spherical invagination in POPC GUVs encapsulating 100 mM sucrose, doped with 1% Rho-DOPE, and subjected to 140 mM glucose. Z-sectioning from vesicle base to apex is shown. Scale bar, 20µm

Video S2. Timelapse of hyperosmotic induced spherical invagination in POPC GUVs encapsulating 100 mM sucrose, doped with 1% Rho-DOPE, subjected to 140 mM glucose. Scale bar, 20 µm

Video S3. Hyperosmotic induced spherical invagination in ternary component GUVs consisting of 32 mol% POPC, 32 mol% Ch, 32 mol% SM, doped with 1 mol% Rho-DOPE (red) and 3 mol% NBD-PE (blue), and encapsulating 100 mM sucrose. Z-sectioning from vesicle base to apex is shown. Scale bar, 20µm

Video S4. Hyperosmotic induced spherical invagination in ternary composition polymer GUVs consisting of 32 mol% PBD_{22}-b- PEO_{14}, 32 mol% POPC, 32 mol% Ch, 1 mol% Rho-DOPE and 3 mol% NBD-PE (blue) encapsulating 100 mM sucrose and subjected to 140 mM glucose. Z-sectioning from vesicle base to apex is shown. Scale bar, 20µm

Video S5. POPC GUVs encapsulating 100 mM sucrose, doped with 1% Rho-DOPE, subjected to an isotonic 100 mM glucose. Z-sectioning from vesicle base to apex is shown. Scale bar, 10 µm

Video S6. Florescence recovery after photobleaching (FRAP) measurement of a spherical invagination of a POPC GUV encapsulating 100 mM sucrose, doped with 1% Rho-DOPE, and subjected to 140 mM glucose. Scale bar, 20 µm

Video S7. Florescence recovery after photobleaching (FRAP) measurement of a spherical invagination of a ternary component GUV consisting of 32 mol% POPC, 32 mol% Ch, 32 mol% SM, doped with 1 mol% Rho-DOPE (red) and 3 mol% NBD-PE (blue), and encapsulating 100 mM sucrose, and subjected to 140 mM glucose. Rho-DOPE is photobleached for fluorescence recovery measurement (shown in the right panel). Scale bar, 20 µm

Video S8. Florescence recovery after photobleaching (FRAP) measurement of a spherical invagination of a POPC GUV encapsulating 100 mM sucrose, doped with 1% Rho-DOPE, subjected to 140 mM glucose (Step 1) and 60 mM glucose (Step 2) bath dilution. Scale bar, 20 µm

Video S9. Phase separation of bud and ternary component mother GUVs consisting of 32 mol% POPC, 32 mol% Ch, 32 mol% SM, doped with 1 mol% Rho-DOPE (red) and 3 mol% NBD-PE (blue), and encapsulating 100 mM sucrose, subjected to 140 mM glucose (Step 1) and 60 mM glucose (Step 2) bath dilution. Scale bar, 20 µm

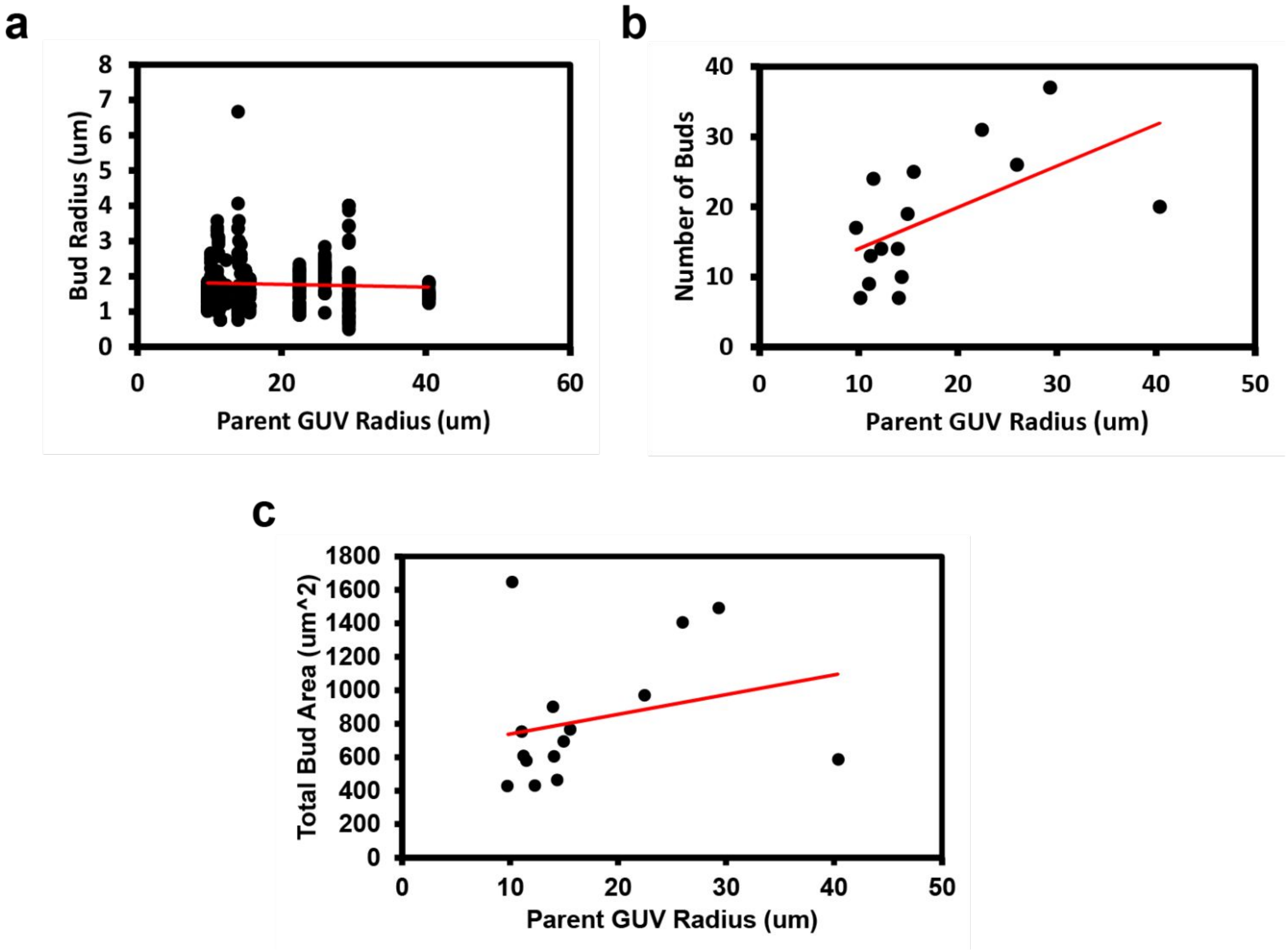

Figure S1. Statistical analysis of hyperosmotic induced spherical invagination in ternary component GUV. (**a-c**) Plots of (a) bud radius (n_{bud}=273, where n_{bud} is number of buds), (b) number of buds (n_{bud}=273), and (c) total bud area as a function of parent GUV radius (n_{GUV}=15 GUVs). The GUV consists of 32 mol% POPC, 32 mol% Ch, 32 mol% SM, 1 mol% Rho-DOPE (red) and 3 mol% NBD-PE (blue).

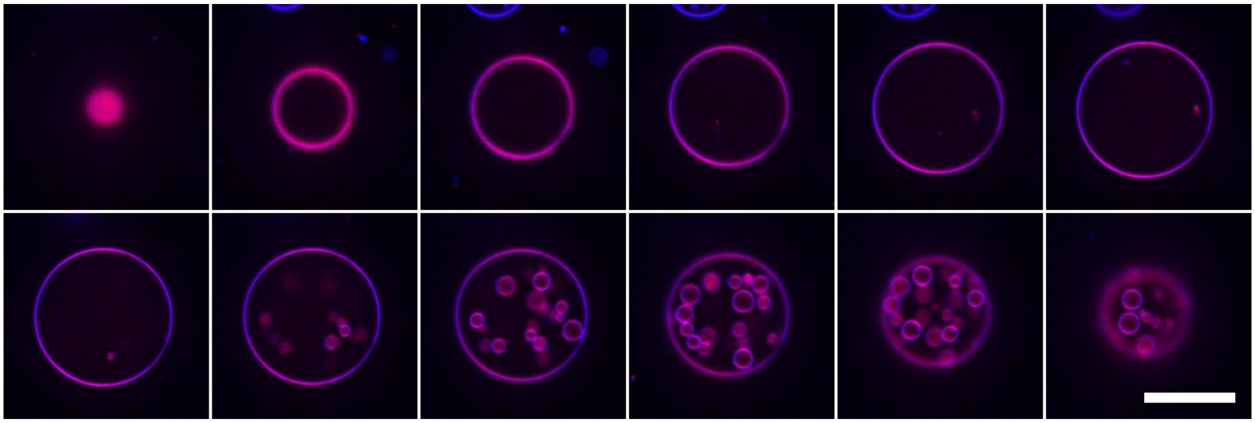

Figure S2. Hyperosmotic induced spherical invagination in ternary component GUVs. The GUVs consist of 32 mol% POPC, 32 mol% Ch, 32 mol% SM, doped with 1 mol% Rho-DOPE (red) and 3 mol% NBD-PE (blue) encapsulating 100 mM sucrose and subjected to 140 mM glucose (n=25). Z-sectioning from vesicle base to apex is shown. Scale bar, 20 µm.

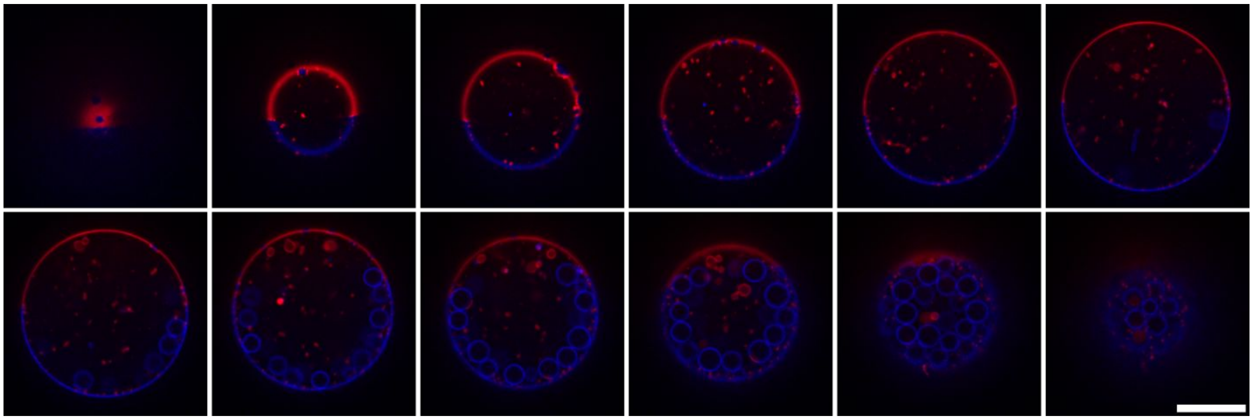

Figure S3. Hyperosmotic induced spherical invagination in ternary component GUVs. The GUVs consist of 32 mol% PBD_{22}-b- PEO_{14}, 32 mol% POPC, 32 mol% Ch, 1 mol% Rho-DOPE and 3 mol% NBD-PE (blue) encapsulating 100 mM sucrose and subjected to 140 mM glucose (n=3). Z-sectioning from vesicle base to apex is shown. Scale bar, 20 µm.

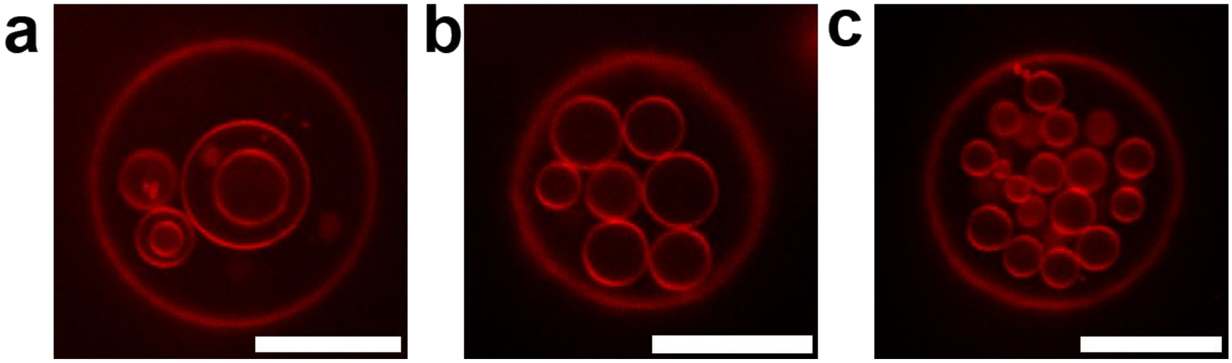

Figure S4. Hyperosmotic induced spherical invagination of single component POPC vesicle at different osmolyte concentration differences (Δc) between vesicle and bath. (**a**) At Δ c =10 mM (n=6). (**b**) At Δ c = 40 mM (n=31). (**c**) At Δ c = 100 mM (n=3). Budding is observed at all instances. Bud in bud structure is prevalent at Δ c =10 mM. Scale bar, 20 µm.

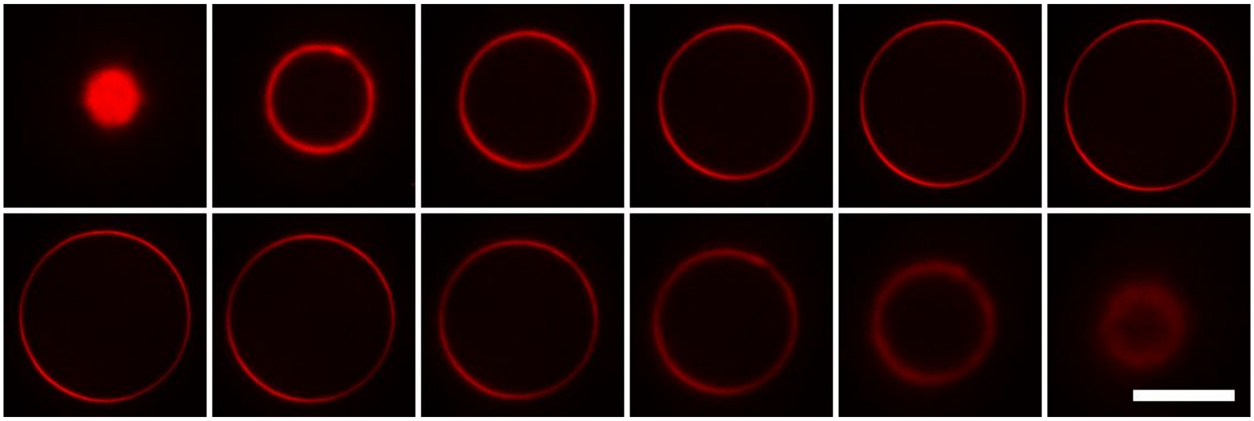

Figure S5. POPC GUVs encapsulating 100 mM sucrose, doped with 1% Rho-DOPE, subjected to an isotonic 100 mM glucose. Z-sectioning from vesicle base to apex is shown (n=3). Scale bar, 20 µm.

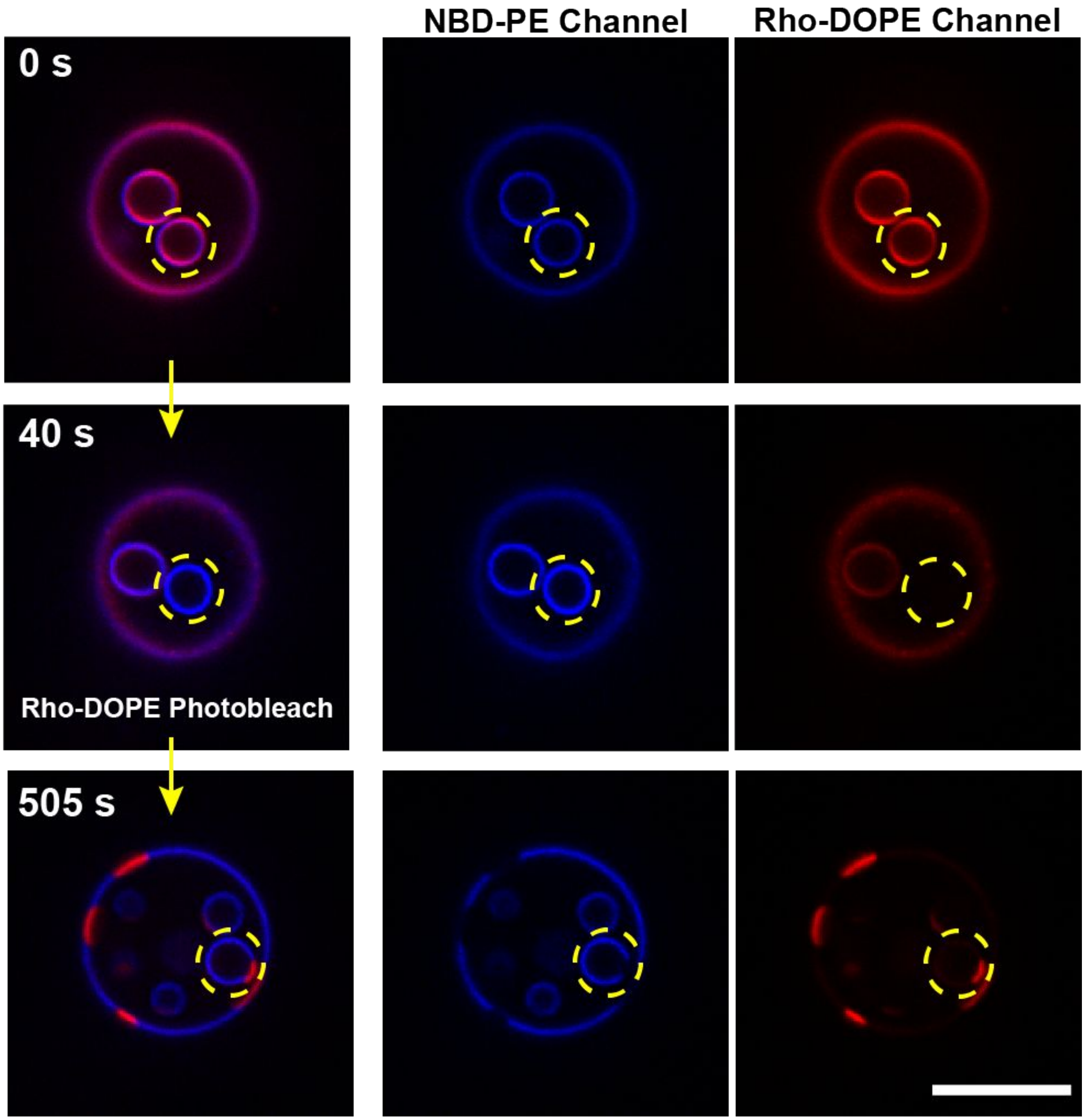

Figure S6: Florescence recovery after photobleaching (FRAP) for a budded ternary component GUV. The GUV consists of 32 mol% POPC, 32 mol% Ch, 32 mol% SM, 1 mol% Rho-DOPE (red) and 3 mol% NBD-PE (blue). Rhodamine labelled lipids are photobleached (yellow dash circle) and fluorescence recovery is observed (n=7). Scale bar, 20 µm.

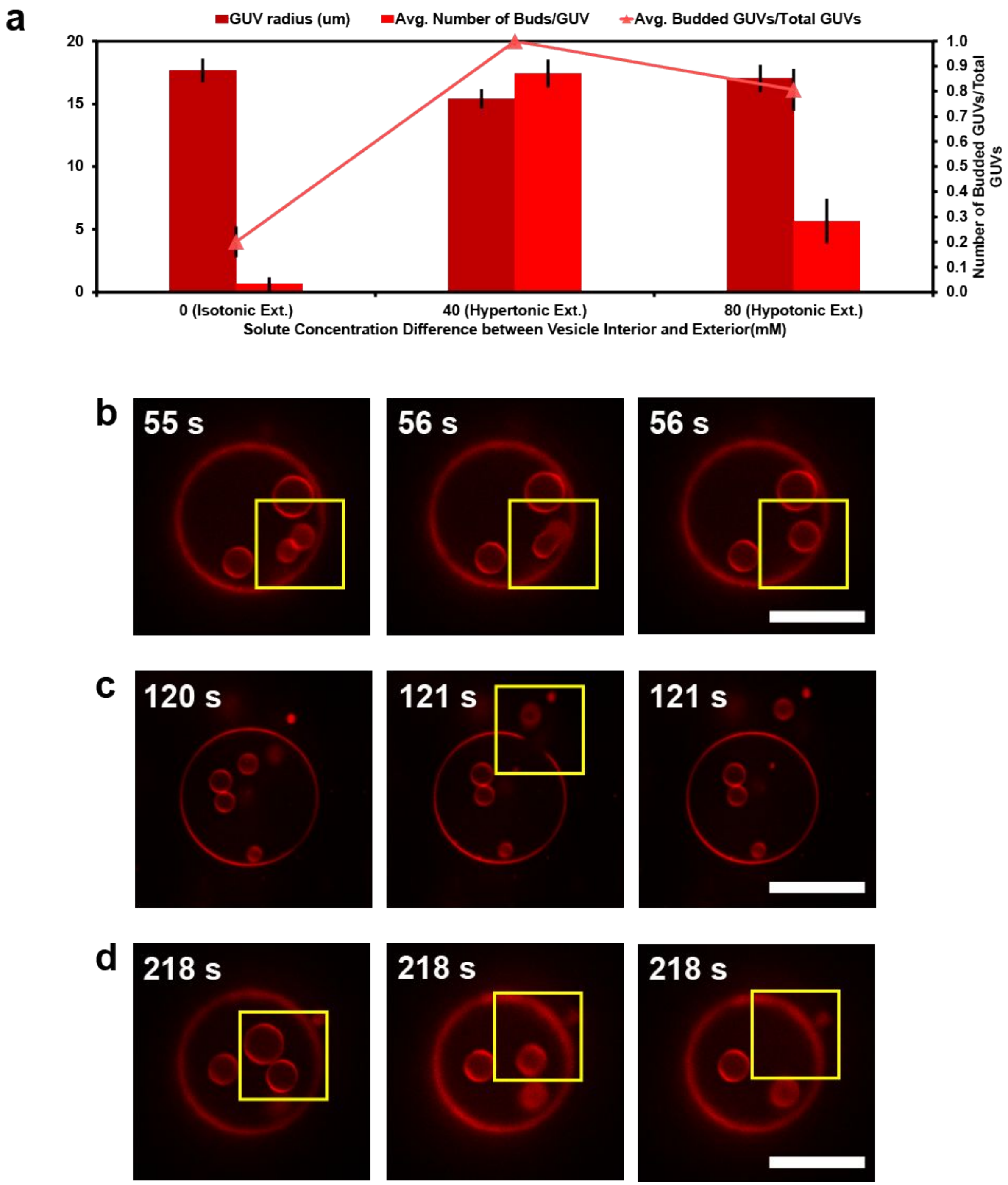

Figure S7: Morphological analysis of budded GUVs subjected to the osmotic cycling process. (**a**) Plots of GUV radius, the average number of buds per GUV, and the average number of budded vesicles over total number of vesicles for POPC GUVs as a function of different solute concentration differentials (n_{GUV}=15 representative GUVs). (**b-d**) Different bud activities (yellow square) after bath dilution (osmotic cycling) (n=3): (**b**) Bud fusion, (**c**) Expulsion of bud through parent transient pore, and (**d**) Bud burst. All scale bars, 20 µm.

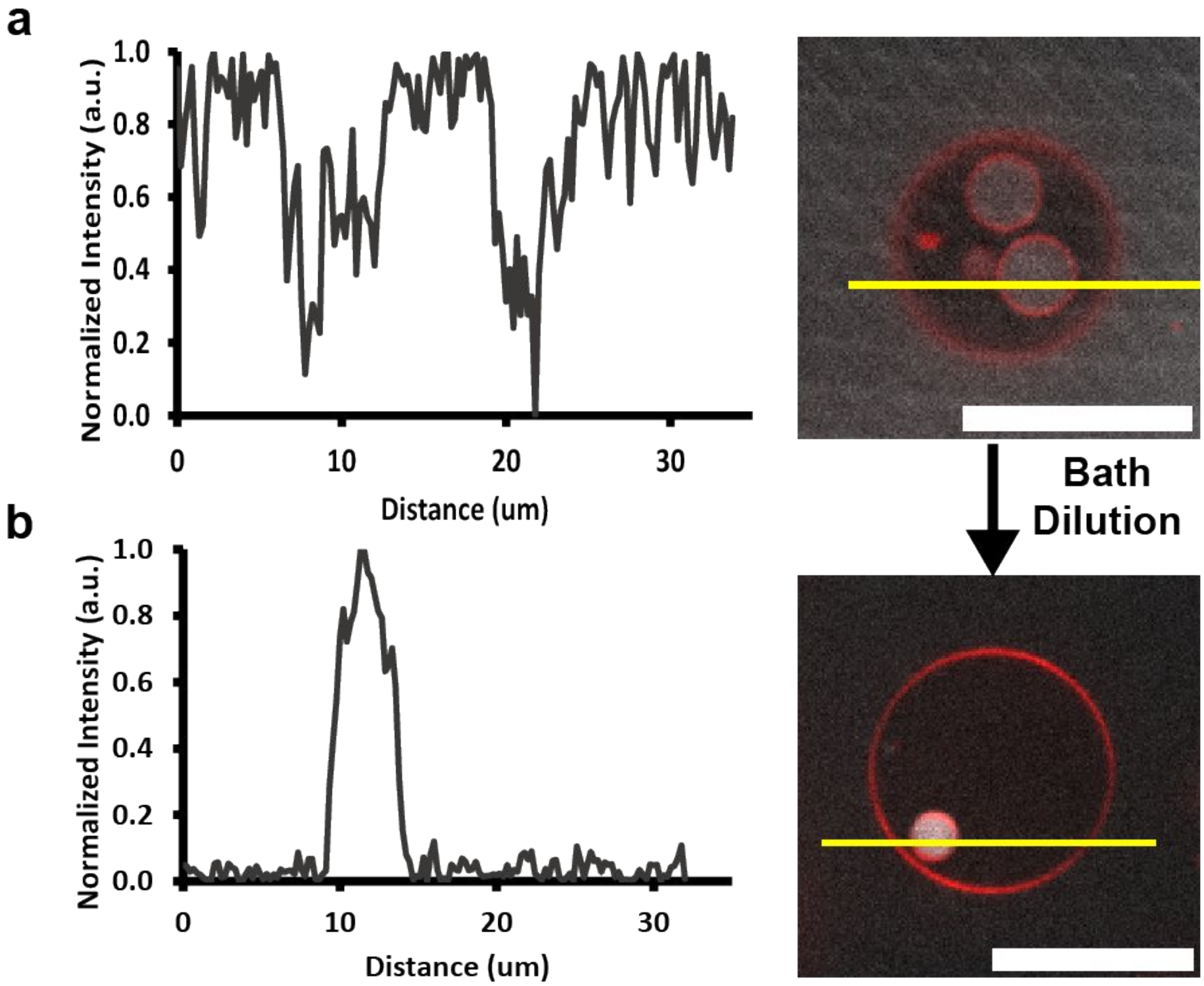

Figure S8: Solute entrapment in buds of ternary component GUVs. The GUV consists of 33 mol% POPC, 33 mol% SM, 33 mol% Ch and 1 mol% Rho-DOPE (red), encapsulating 100 mM sucrose, and subjected to the two-step osmotic cycle using fluorescently doped (0.1 mM NBD-glucose) glucose solution (blue) in the exterior bath. (**a**) After hyperosmotic stress (Step 1) (n=7). (**b**) After hypotonic stress (Step 2) (n=8). Plots of intensity profiles (*left panel*) of the yellow lines (*right panel*) overlaid on representative fluorescence micrographs. Scale bar, 20 µm.

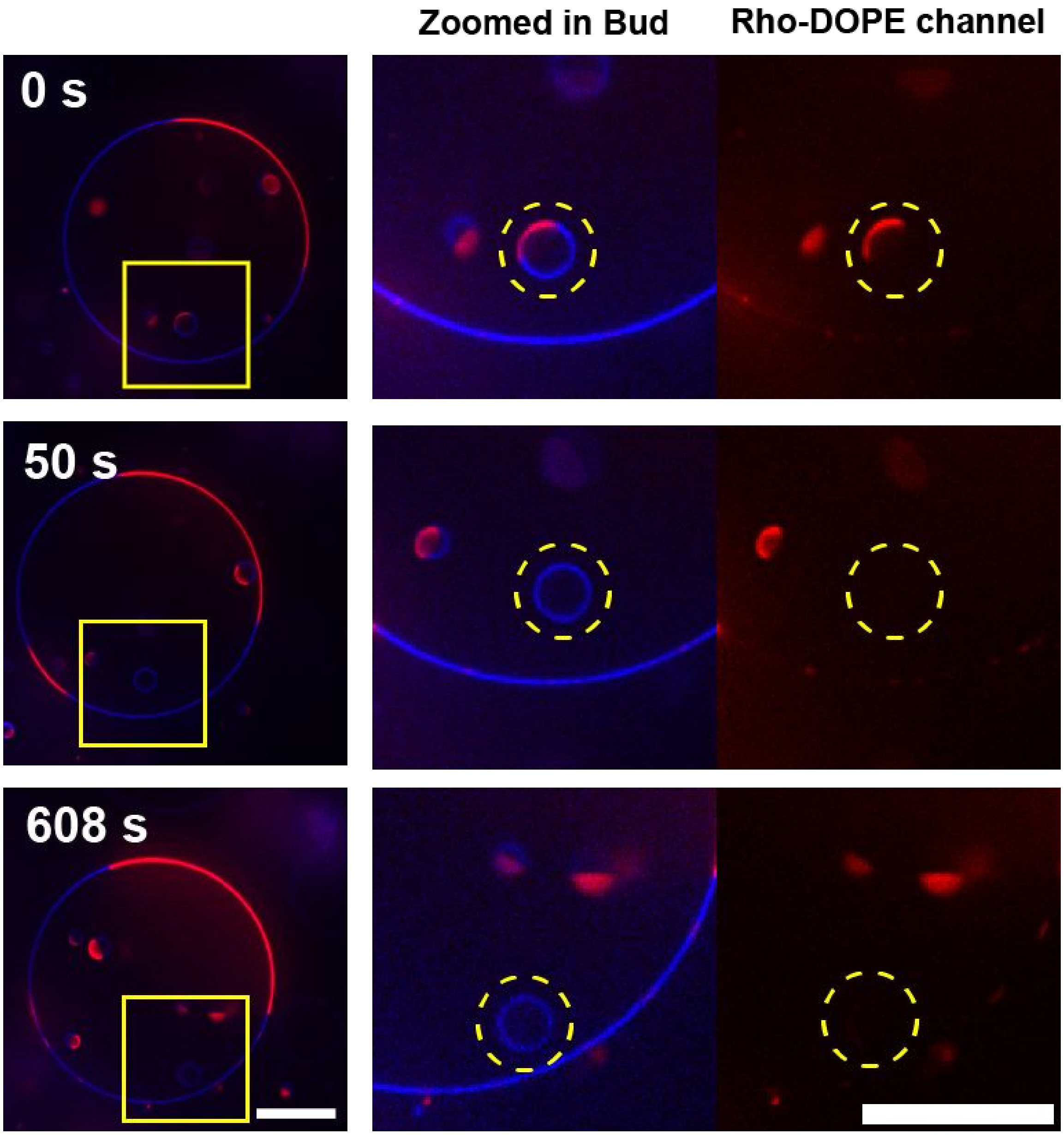

Figure S9: Florescence recovery after photobleaching (FRAP) for a budded ternary component GUV after osmotic cycling. The GUV consists of 32 mol% POPC, 32 mol% Ch, 32 mol% SM, 1 mol% Rho-DOPE (red) and 3 mol% NBD-PE (blue). Rhodamine labelled lipids are photobleached (yellow dash circle) and fluorescence recovery is not observed (n=3). Scale bar, 20 µm.

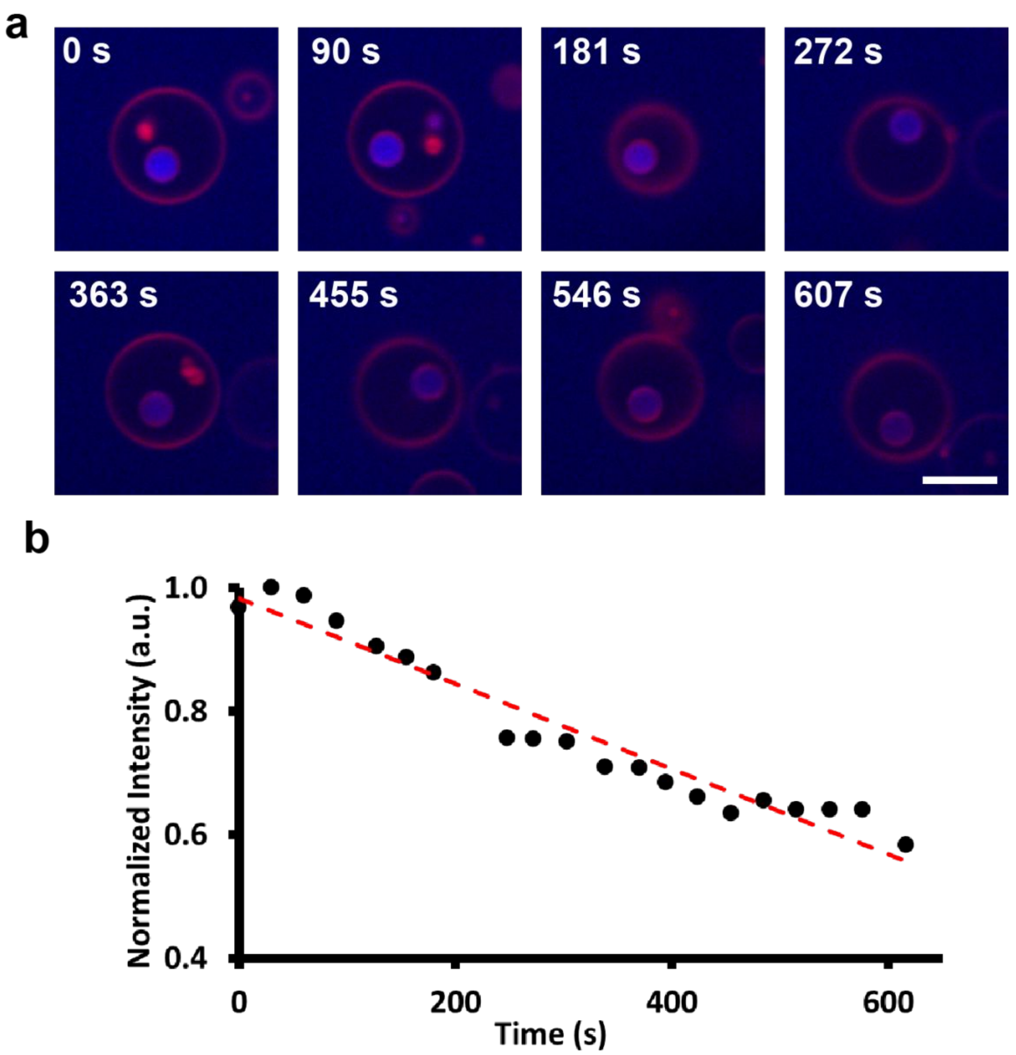

Figure S10: Osmotic cycling of POPC GUVs. a) Selected frames from a time-lapse series of POPC GUVs subjected to the two-step osmotic cycling process. The GUV consists of 99 mol% POPC and 1 mol% Rho-DOPE (red), subjected to the two-step osmotic cycle using fluorescently doped (0.1 mM NBD-glucose) glucose solution (blue) in the exterior bath (n=4). Scale bar, 10 µm. (b) Plot of normalized florescence intensity of bud interior as a function of time.
